# Pity the Sisters

**Published:** 1874-01

**Authors:** 


### PITY THE SISTERS.

It is hard enough for men to make a living these times, but how much harder for poor women, who are cut off from so many of the occupations for which men are adapted. There are thousands of struggling sisters, and daughters, and mothers in New York to-day bravely battling against poverty, and want, and absolute destitution, hungering in cheerless rooms, shivering in crazy garrets, or slowly dying in cold, damp cellars, because they have not the means to procure better lodgings; and yet, in these countless cases of want, and suffering, and sorrow, they hold fast their integrity, and in virtuous living, struggle on.

There are various institutions in New York, under the management of women of culture and refinement and high social position, who spend a large share of their time in endeavoring to procure remunerative work for those who are willing to do it, and who are more than thankful to get something to do by which they can earn the means to procure a loaf of bread—aye, a single "roll," at the baker's. Among the other commendable institutions is "The Free Labor Bureau," whose object is to procure work for both men and women. In the three weeks ending October 18th, places have been found for 183 men and 1,760 women. Nearly 600 women a week snatched from suffering and terrible temptation, and placed in comfortable and self-supporting situations. But money is needed to carry on a work of this kind ; a large house has to be rented to give shelter to those who are waiting their turn, and food, and fuel, and bedding must be had; and while generous hearted persons are giving their time in hunting out and procuring places, there are many who cannot. Is it too much to ask the reader, who may be among this latter class, to do something in the way of sending money, even if it be but a dollar, and how much better if it should be ten? That ten dollars would procure ten places for ten poor young girls, who would have a plenty to eat every day, and comfortable, and warm, and tidy places to work in. But do our country readers feel as they ought their individual obligations to send their subscriptions to the city? Will you allow New Yorkers to do all the work and furnish the means, when a very large number of all the destitute were born in the country, have come up to the great city from the farms and villages of the land to try their fortunes, and have failed? Let it be remembered by all who wish to make safe investments in these perilous times, that "He who giveth to the poor lendeth to the Lord," who always pays high "interest," and who never had a note "protested."

